# Sensitive and Specific Recombinase Polymerase Amplification Assays for Fast Screening, Detection, and Identification of Bacillus anthracis in a Field Setting

**DOI:** 10.1128/AEM.00506-18

**Published:** 2018-05-17

**Authors:** Mostafa Bentahir, Jérôme Ambroise, Cathy Delcorps, Paola Pilo, Jean-Luc Gala

**Affiliations:** a Centre de Technologies Moléculaires Appliquées, Institut de Recherche Expérimentale et Clinique, Université catholique de Louvain, Brussels, Belgium; b Biothreats Unit, Defense Laboratories Department, Belgian Armed Forces, Brussels, Belgium; c Institute of Veterinary Bacteriology, Vetsuisse, University of Bern, Bern, Switzerland; Goethe University Frankfurt am Main

**Keywords:** biothreat agents, isothermal amplification, quantitative PCR

## Abstract

Four isothermal recombinase polymerase amplification (RPA) assays were developed for fast in-field identification of Bacillus anthracis. The RPA assays targeted three specific sequences (i.e., the BA_5345 chromosomal marker, the lethal factor *lef* [from pXO1], and the capsule-biosynthesis-related *capA* [from pXO2]) and a conserved sequence in the adenylate cyclase gene (*adk*) for the Bacillus cereus group. B. anthracis-specific RPA assays were tested first with purified genomic DNAs (*n* = 60), including 11 representatives of B. anthracis, and then with soil (*n* = 8) and white powder (*n* = 8) samples spiked with inactivated B. anthracis spores and/or other biological agents. The RPA assays were also tested in another laboratory facility, which blindly provided DNA and lysate samples (*n* = 30, including 20 B. anthracis strains). RPA assays displayed 100% specificity and sensitivity. The hands-off turnaround times at 42°C ranged from 5 to 6 min for 10^2^ genomic copies. The analytical sensitivity of each RPA assay was ∼10 molecules per reaction. In addition, the BA_5345 and *adk* RPA assays were assessed under field conditions with a series of surface swabs (*n* = 13, including 11 swabs contaminated with B. thuringiensis spores) that were blindly brought to the field laboratory by a chemical, biological, radiological, and nuclear (CBRN) sampling team. None of the 13 samples, except the control, tested positive for B. anthracis, and all samples that had been harvested from spore-contaminated surfaces tested positive with the *adk* RPA assay. All three B. anthracis-specific RPA assays proved suitable for rapid and reliable identification of B. anthracis and therefore could easily be used by first responders under field conditions to quickly discriminate between a deliberate release of B. anthracis spores and a hoax attack involving white powder.

**IMPORTANCE** In recent decades, particularly following the 11 September 2001 and Amerithrax attacks, the world has experienced attempts to sow panic and chaos in society through thousands of white-powder copycats using household powders to mimic real bioterrorism attacks. In such circumstances, field-deployable detection methods are particularly needed to screen samples collected from the scene. The aim is to test the samples directly using a fast and reliable assay for detection of the presence of B. anthracis. While this would not preclude further confirmatory tests from being performed in reference laboratories, it would bring useful, timely, and relevant information to local crisis managers and help them make appropriate decisions without having to wait for quantitative PCR results (with turnaround times of a few hours) or phenotypic identification and sequencing (with turnaround times of a few days). In the current investigation, we developed a set of isothermal RPA assays for the rapid screening and identification of B. anthracis in powders and soil samples, with the purpose of discriminating a deliberate release of B. anthracis spores from a hoax attack involving white powder; this would also apply to dispersion by spraying of aerosolized forms of B. anthracis. Further work is now ongoing to confirm the first observations and validate the on-site use of these assays by first responders.

## INTRODUCTION

Bacillus anthracis, the etiological agent of anthrax, is a Gram-positive endospore-forming bacterium that can cause a life-threatening disease in livestock and occasionally in humans (1). This zoonotic pathogen exerts its virulence activity through pXO1 and pXO2 plasmids carrying unique genes responsible for toxin production and capsule synthesis, respectively (2–4). Intentional dispersion of B. anthracis spores for bioterroristic purposes or in the context of military operations constitutes a major threat for both civilians and ground troops. In recent decades, particularly following the 11 September 2001 and Amerithrax attacks, the world has experienced several attempts to sow panic and chaos in society through deliberate dispersion by thousands of white-powder copycats using household powders to mimic real bioterrorism attacks (5–7). Under such circumstances, rapid and reliable detection and identification methods are particularly needed to quickly screen samples collected from the scene, i.e., to test directly for the presence of B. anthracis. Conventional cultivation methods are clearly inappropriate under such conditions, because they depend on highly skilled experts, require specific biosafety level 3 (BSL 3) facilities, and have turnaround times of several hours or days (8). The high specificity, sensitivity, and speed of nucleic acid molecular methods, including real-time quantitative PCR (qPCR), explain why these methods have become a predominant diagnostic tool (9, 10). In addition, novel DNA-based technologies based on isothermal amplification are now steadily gaining interest among first responders, because of their simplicity, speed, and appropriateness for in-field use. Isothermal amplification enables health care workers in remote locations to quickly analyze samples for the presence of nucleic acids from a range of infectious agents, using field-deployable assays (11–15). Among these methods, loop-mediated isothermal amplification (LAMP) (16) and recombinase polymerase amplification (RPA) (17) assays have become increasingly popular, and they are now proposed in the setting of bioterrorism for rapid detection of biothreats, including B. anthracis (18–20).

The major challenge for developing a B. anthracis-specific detection assay stems from the close genetic relationships among a cluster of strains referred to as the Bacillus cereus sensu lato group. This cluster comprises B. anthracis, B. cereus, Bacillus thuringiensis, Bacillus mycoides, Bacillus pseudomycoides, Bacillus weihenstephanensis, and Bacillus cytotoxicus (21–23). The degrees of genetic similarity are substantially higher among B. anthracis, B. cereus, and B. thuringiensis, compared to the other species; some authors even proposed considering them a single species (24). In that respect, the phylogenetic and taxonomic relationships of the bacteria in the B. cereus group are still under intense discussion (25). To identify B. anthracis, PCR assays commonly amplify target sequences from the pXO1 and pXO2 virulence plasmids (10, 22). However, nonpathogenic B. anthracis isolates lacking these virulence plasmids, as well as a few B. cereus strains harboring anthrax-like plasmids, have been characterized (6, 26). These findings underscore the need for assays targeting unique chromosomal signatures combined with plasmid genes, to distinguish B. anthracis isolates from closely related B. cereus group isolates and fully pathogenic B. anthracis strains from attenuated or nonvirulent B. anthracis strains (27). However, detecting B. anthracis chromosomal signature sequences has proved to be very challenging. Among all reported targets, only a few, e.g., *purA* and *pclR* single-nucleotide polymorphisms (SNPs), as well as BA_5345, *PL3*, and BA5357 genes, were found to be unique to B. anthracis (9, 25).

**FIG 1:**
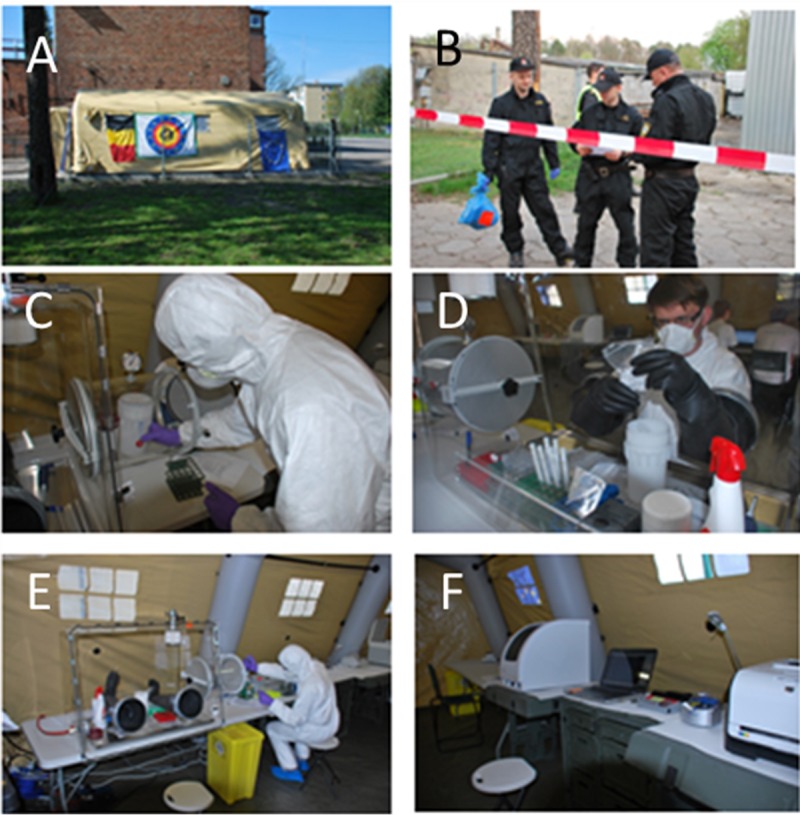
Main steps carried out in a laboratory tent in the field for sample processing from receipt to the final analyses. The laboratory tent was deployed in the field (A), and samples were brought to the laboratory by the Polish State Firemen CBRN sampling team (B). Samples were processed in a glove box to inactivate potential bioagents present in the sample (C and D), and nucleic acids were then extracted outside the glove box (E). qPCR and RPA assays were carried out, and data were analyzed (F).

Here we report the design and validation of a set of three isothermal RPA assays that allow fast and specific screening and identification of B. anthracis in powders and soil samples. The assays amplify the BA_5345 chromosomal marker and the *lef* (pXO1) and *capA* (pXO2) virulence genes. The first validation was carried out on a well-characterized DNA collection of B. anthracis and B. cereus group members, as well as DNA strains outside the B. cereus group. This validation was completed by blind testing in an external laboratory facility, which provided well-characterized DNA samples from its own collection of B. anthracis and B. cereus group members. Optimized assays were subsequently used for detection of B. anthracis in powder and soil samples spiked with inactivated B. anthracis spores and/or other biological agents. A fourth RPA assay targeted a conserved sequence of the *adk* gene, which is shared by all representatives of the B. cereus group. Finally, BA_5345 and *adk* RPA assays were evaluated under field conditions (Fig. 1) by processing samples with and without B. thuringiensis, a widely used simulant of B. anthracis. Data were compared to those obtained with our previously validated duplex qPCR assay (28).

## RESULTS

### RPA assay optimization.

**FIG 2:**
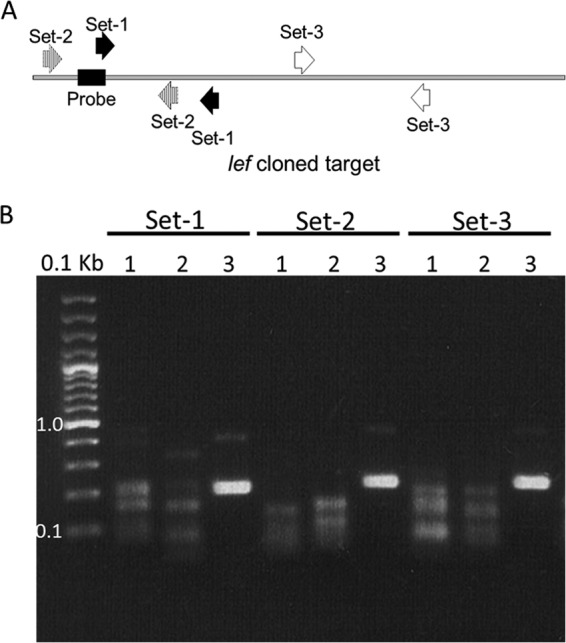
RPA assay optimization. Optimization was achieved using the TwistAmp Basic amplification kit, as illustrated for the *lef* target gene. (A) Relative positions of RPA primer pairs (sets 1 to 3) used in screening and the part of the target where the *lef* probe was designed. (B) Results of gel electrophoresis analysis following target amplification with the indicated sets of primers. Samples 1 and 2 were NTC samples prepared in pre-PCR and post-PCR rooms, respectively, while sample 3 contained the target DNA.

In the first optimization phase using the TwistAmp Basic amplification kit, three pairs of RPA primers, targeting various sequence regions of BA_5345, *lef*, *capA*, and *adk* cloned target genes, were selected based on the highest amplification efficiency and lowest background amplification in gel electrophoresis (Fig. 2). Unlike the set 1 and set 3 pairs of primers, the *lef* sequence region targeted by the set 2 pair of RPA primers matched these criteria (Fig. 2A). A similar approach was used to select the optimal target region within the BA_5345, *capA*, and *adk* sequences.

After selection of the optimal target regions, optimization was carried out using the real-time fluorescent DNA amplification TwistAmp exo kit. RPA primers and probe were selected according to the greatest and fastest fluorescence amplification and negative no-template control (NTC) signals (Table 1). The optimal temperature of the RPA reaction was set at 42°C. Following an initial preincubation for 3 min, the tubes were removed from the thermoblock, mixed vigorously, centrifuged at 5,000 rpm for 10 s, and incubated for the remaining time. Using 5 × 10^2^ genomic copies of each BA_5345, *lef*, *capA*, and *adk* cloned target under optimal reaction conditions, the detection threshold times were 5.3, 6.3, 4.7, and 5.3 min, respectively. RPA results with pXO1 (*lef*) and pXO2 (*capA*) targets were concordant with those obtained with our duplex qPCR assay designed to detect the same plasmid sequences (28).

**TABLE 1:** Primers and probes used in this study

Type and target	Name	Sequence (5′ to 3′)^*a*^	Starting position	Ending position	Size (bases)	Amplicon size (bp)
PCR primers						
*lef*	lef-For	CGCTTCATTTGTTCTCCCATAC	139956	139935	22	860
	lef-Rev	CAACCCTAGGTGCGGATTTAG	139097	139117	21	
*capA*	capA-For	GGTACAACGTACAGAAGCAGT	18512	18532	21	971
	capA-Rev	GAGCACCCTTGGATGTATCTTT	19482	19461	22	
BA_5345	BA-For	CGATTTTGTGGATTGCGTATG	4873856	4873876	21	493
	BA-Rev	ACCGCAAGTTGAATAGCAAG	4874348	4874329	20	
*adk*	adk-For	CCGAACAGATTGTTGCCAAG	4360272	4360253	20	600
	adk-Rev	ACGCTAAGCCTCCGATGAGA	4359673	4359692	20	
RPA primers						
*lef*	lef-58-For	TTAGAATTTGTAACTAAATCAGATTGGTTCT	139889	139859	31	146
	lef-60-Rev	CGTTCTATATTACTCCATGGACCTTCAAA	139744	139772	29	
*capA*	capA-45-For	CGGATTATGGTGCTAAGGGAACTAAAGATAC	18838	18868	31	145
	CapA-63-Rev	CCAAGAGTAGCAACCCTAACACCATTTAC	18982	18954	29	
BA_5345	BA-31-For	GTCTGGCACATGGTACTACTCAAACAAGAT	4874134	4874163	30	105
	BA-36-Rev	GAACAATGACCCTAGTGCATGTGTAGTTCC	4874238	4874209	30	
*adk*	adk-27-For	GTGTGCGATAAATGTGGTGGCGAATTATATCAAC	4359877	4359844	34	137
	adk-28-Rev	CTTTGTAGGTAACCAAGCTCCTCGTAGAAATCAAG	4359741	4359775	35	
Probes						
*lef*	lef-exo-55	AATTTGTAACTAAATCAGATTGGTTCT**T**A**T**C**T**AATAGATATCCAG	139885	139841	45	
*capA*	capA-exo-56	AAGGCCTTTAAAGAAGCTGATCTTGAC**T**A**T**G**T**GGGTGCTGGTGAA	18873	18917	45	
BA_5345	BA-exo-54	CTCAAACAAGATTCAGAGACTCGTACA**T**A**C**A**T**AGAAGGACGATAC	4874152	4874196	45	
*adk*	adk-exo-53	TGATGACAATGAAGAAACTGTAGCAAA**T**C**G**C**T**TAGATGTAAATATTA	4359839	4359793	47	

a Bases in bold indicate where dT-FAM, THF, and dT-BHQ1, respectively, are inserted.

### RPA assay sensitivity and speed.

**FIG 3:**
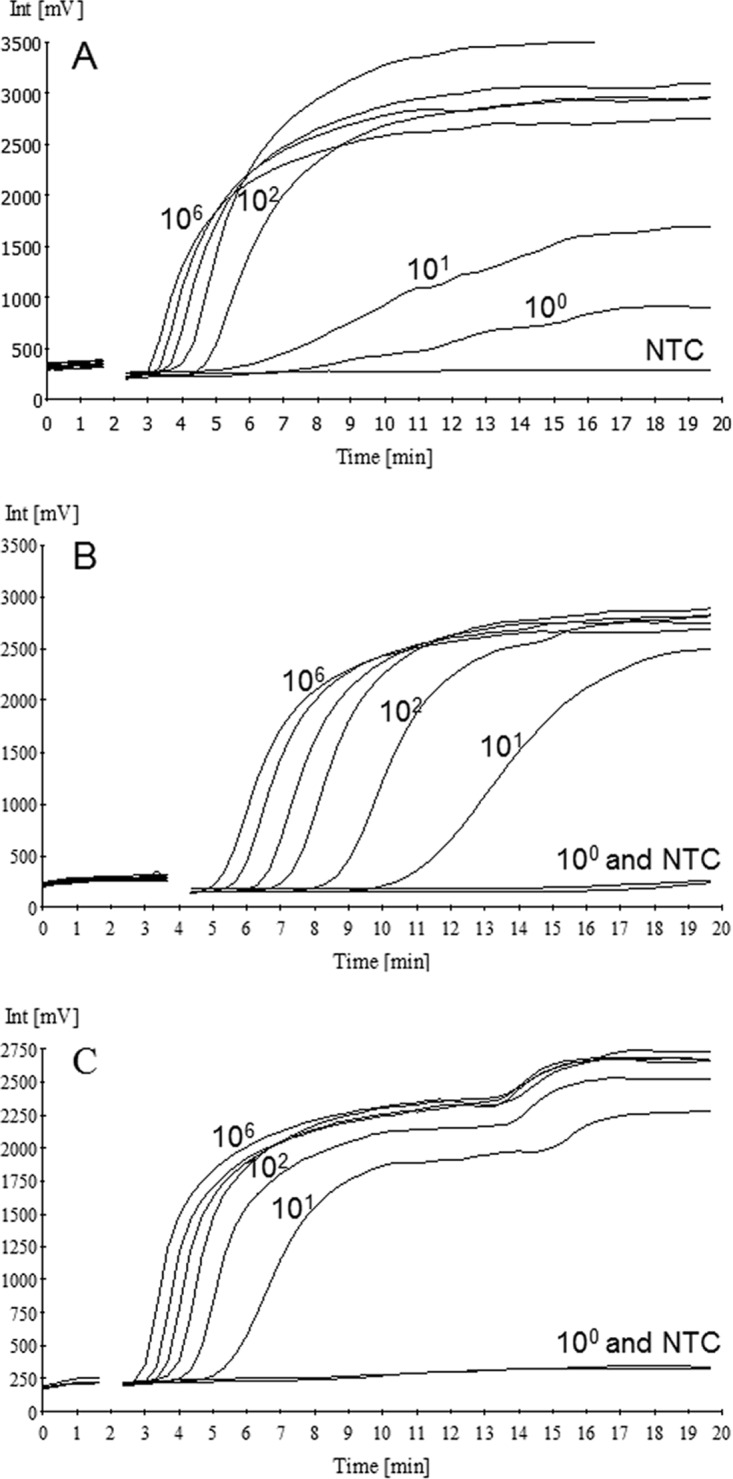
RPA plots. Amplification was carried out with 10-fold serial dilutions of BA_5345 (A), *lef* (B), and *capA* (C) target sequences, as well as NTC samples.

**FIG 4:**
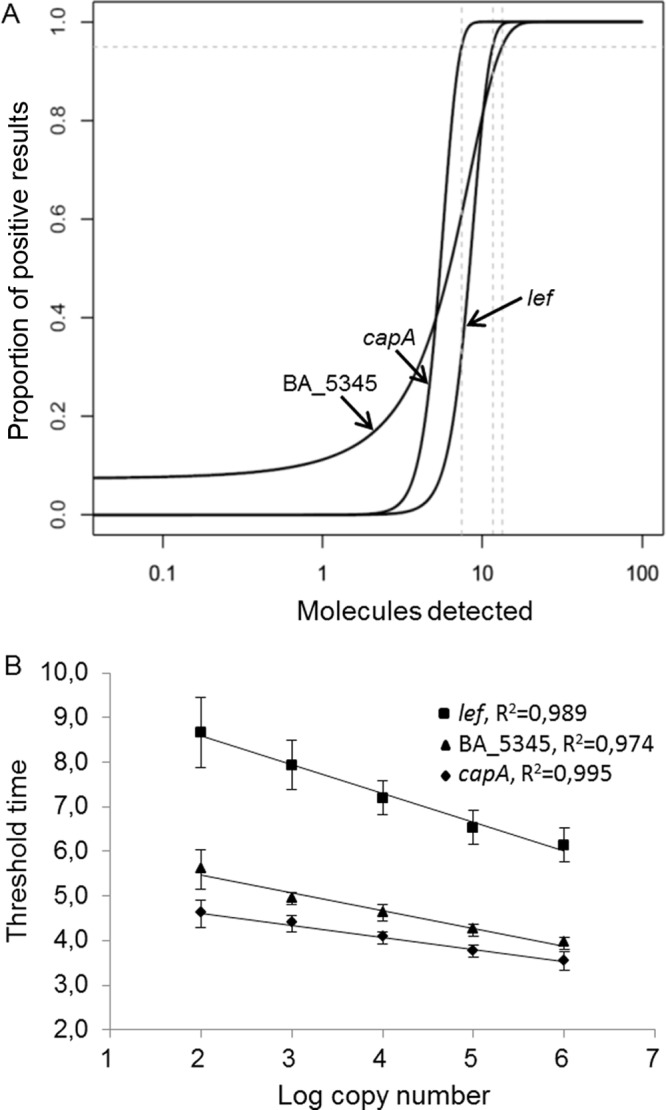
Analytical sensitivity and quantitative ranges of BA_5345, *lef*, and *capA* RPA assays. (A) LOD values, as determined by probit regression analysis. (B) Assay linearity ranges.

Analytical sensitivity was assessed under optimal conditions with 10-fold serial dilutions of recombinant plasmid standards carrying the BA_5345, *lef*, or *capA* targets. A recombinant a*dk* plasmid was not included in this assessment. For each RPA, the concentration target of the DNA measured by fluorescence covered 5 orders of magnitude (Fig. 3A, B, and C). The limit of detection (LOD) values for each RPA at a 95% detection probability, as estimated by a probit regression analysis of quintuplicate tests, were 13.31, 11.61, and 7.43 molecules per reaction for BA_5345, *lef*, and *capA*, respectively (Fig. 4A). The RPA reactions showed excellent linearity over a dynamic range of 10^2^ to 10^6^ copies for the BA_5345, *lef*, and *capA* targets, with *R*^2^ correlation coefficients of 0.97, 0.98, and 0.99, respectively (Fig. 4B).

BA_5345, *lef*, and *capA* RPA assays correctly identified B. anthracis reference DNAs (*n* = 11), powders containing a fixed concentration of B. anthracis Sterne spores (*n* = 4), and soil samples spiked with different concentrations of B. anthracis (Ames strain) spores (*n* = 3), with 100% sensitivity (*n* = 18). The mean detection times for BA_5345, *lef*, and *capA* using B. anthracis reference DNAs were 4.37, 6.80, and 4.23 min, respectively. Data from three replicates were reproducible. It is of note that powder samples spiked with B. anthracis spores were BA_5345 and *lef* positive and *capA* negative, in accordance with the B. anthracis Sterne strain (pXO1 positive and pXO2 negative).

### RPA assay specificity and cross-reactivity.

Specificity was evaluated with a panel of 50 reference DNAs from strains phylogenetically distant from or very close to B. anthracis, 4 negative powder samples, 5 soil samples spiked with single or multiple biological agents, and 13 swab samples (with [*n* = 11] or without [*n* = 2] B. thuringiensis spores) tested under field conditions (Tables 2 and 3). Pooled results from all samples tested with the RPA assay (*n* = 72) indicated an overall specificity of 100%. No nonspecific signal was generated with 50 non-B. anthracis strains, including closely related B. cereus group strains, Bacillus sp. strains, and bacteria frequently encountered as contaminants during sampling. No background signal was detected with human DNA. No cross-detection was observed for the samples containing either B. thuringiensis or other bacterial or viral biological agents.

**TABLE 2:** Bacterial strains used to test RPA assay specificity and sensitivity

Species^*a*^	Original identification	Source^*b*^	RPA detection time (min)			qPCR results^*c*^		
			BA_5345	*lef* (pXO1)	*capA* (pXO2)	*adk*	pXO1	pXO2
B. anthracis	9508	CEB	4.57 ± 0.23	6.80 ± 0.17	4.30 ± 0.00	+	+	+
B. anthracis	9531	CEB	4.20 ± 0.17	−	−	+	−	−
B. anthracis	9774	CEB	4.53 ± 0.40	6.23 ± 0.40	4.77 ± 0.40	+	+	+
B. anthracis	9506	CEB	4.10 ± 0.17	6.10 ± 0.17	4.67 ± 0.35	+	+	+
B. anthracis	9534	CEB	4.10 ± 0.17	−	4.67 ± 0.35	+	−	+
B. anthracis	9439	CEB	4.20 ± 0.17	6.53 ± 0.40	−	+	+	−
B. anthracis	9602	CEB	4.43 ± 0.51	6.43 ± 0.23	4.43 ± 0.23	+	+	+
B. anthracis	9440	CEB	4.30 ± 0.00	8.20 ± 0.17	4.57 ± 0.23	+	+	+
B. anthracis	VAR06/7570.4CAP	UCL	4.57 ± 0.23	7.00 ± 0.30	3.57 ± 0.23	+	+	+
B. anthracis	VAR06/1106.3#2	UCL	4.57 ± 0.23	6.90 ± 0.17	3.43 ± 0.23	+	+	+
B. anthracis	VAR06/5348.3#1	UCL	4.70 ± 0.00	7.00 ± 0.00	3.67 ± 0.35	+	+	+
B. cereus	ATCC 14579	UCL	−	−	−	+	−	−
B. cereus	ATCC 10987	UCL	−	−	−	+	−	−
B. cereus	DSM 2302	UCL	−	−	−	+	−	−
B. cereus	DSM 345	BW	−	−	−	+	−	−
B. cereus	S3A	CTMA	−	−	−	+	−	−
B. cereus	ATCC 13061 (HT1-A1)	UCL	−	−	−	+	−	−
B. cereus	ATCC 10876 (HT1-A2)	UCL	−	−	−	+	−	−
B. cereus	ATCC 21282 (HT1-C1)	UCL	−	−	−	+	−	−
B. mycoides	MYC005	UCL	−	−	−	+	−	−
B. mycoides	ATCC 6463	UCL	−	−	−	+	−	−
B. mycoides	MYC003	UCL	−	−	−	+	−	−
B. mycoides	WSBC 10211	BW	−	−	−	+	−	−
B. mycoides	HBS 1-16	UCL	−	−	−	+	−	−
B. pseudomycoides	NRRL B-617	UCL	−	−	−	+	−	−
B. pseudomycoides	NRRL BD5	UCL	−	−	−	+	−	−
B. pseudomycoides	NRRL NRS 321N	UCL	−	−	−	+	−	−
B. pseudomycoides	6.A.3	CTMA	−	−	−	+	−	−
B. weihenstephanensis	WSBC 28005	BW	−	−	−	+	−	−
B. weihenstephanensis	WSBC 10204	UCL	−	−	−	+	−	−
B. weihenstephanensis	WSBC 10278	BW	−	−	−	+	−	−
B. weihenstephanensis	WSBC 10207	UCL	−	−	−	+	−	−
B. weihenstephanensis	WS2481	UCL	−	−	−	+	−	−
B. weihenstephanensis	WSBC 10201	UCL	−	−	−	+	−	−
B. thuringiensis	4Q2-72	UCL	−	−	−	+	−	−
B. thuringiensis	T03A016 = HD1	UCL	−	−	−	+	−	−
B. thuringiensis	WSBC 10206	BW	−	−	−	+	−	−
B. thuringiensis	ABTS-1857	CTMA	−	−	−	+	−	−
B. thuringiensis	HD73	UCL	−	−	−	+	−	−
B. thuringiensis	Bt5	UCL	−	−	−	+	−	−
B. thuringiensis	ATCC 39646	UCL	−	−	−	+	−	−
Bacillus subtilis	ATCC 6633	UCL	−	−	−	−	−	−
B. subtilis	ATCC 12711	UCL	−	−	−	−	−	−
B. subtilis	ATCC 6051	UCL	−	−	−	−	−	−
B. subtilis	168 (Suxia)	UCL	−	−	−	−	−	−
B. subtilis	DSMZ 5934	CTMA	−	−	−	−	−	−
Bacillus amyloliquefaciens	ATCC 23350	UCL	−	−	−	−	−	−
Bacillus badius	ATCC 14574	UCL	−	−	−	−	−	−
Bacillus sphaericus	ATCC 10208	UCL	−	−	−	−	−	−
Bacillus atrophaeus	ATCC 9372	CTMA	−	−	−	−	−	−
Streptococcus pyogenes	ATCC 12344	CTMA	−	−	−	−	−	−
Enterococcus faecalis	ATCC 29212	CTMA	−	−	−	−	−	−
Stenotrophomonas maltophilia	ATCC 13637	CTMA	−	−	−	−	−	−
Escherichia coli	DSMZ 8579	CTMA	−	−	−	−	−	−
Enterobacter aerogenes	DSMZ 30053	CTMA	−	−	−	−	−	−
Serratia liquefaciens	ATCC 27592	CTMA	−	−	−	−	−	−
Staphylococcus aureus	NCTC 10442	CTMA	−	−	−	−	−	−
Streptococcus pneumoniae	DSMZ 20566	CTMA	−	−	−	−	−	−
Klebsiella pneumoniae	ATCC 13883	CTMA	−	−	−	−	−	−
Clostridium difficile	DSMZ 1296	CTMA	−	−	−	−	−	−

a After extraction and measurement of DNA concentrations, 5 pg was used in each RPA reaction. Samples with negative results were amplified in parallel using a 16S rRNA PCR.

b CEB, Centre d'Etudes du Bouchet (France); UCL, Université catholique de Louvain (Belgium); BW, Bundeswehr (Germany); CTMA, Centre de Technologies Moléculaires Appliquées (Belgium).

c −, not detected; +, detected.

**TABLE 3:** Environmental powder and soil samples (NATO SIBCRA exercise)

Sample no.^*a*^	Matrix	Spiking status	Concentration^*b*^	RPA results^*d*^		
				BA_5345	*lef* (pXO1)	*capA* (pXO2)
1^*c*^	DiPel powder	B. thuringiensis	ND	−	−	−
2	DiPel powder	B. thuringiensis plus B. anthracis Sterne	ND	+	+	−
3^*c*^	Backing soda	None	ND	−	−	−
4	Backing soda	B. anthracis Sterne	ND	+	+	−
5^*c*^	Yeast powder	None	ND	−	−	−
6	Yeast powder	B. anthracis Sterne	ND	+	+	−
7^*c*^	Cream powder	None	ND	−	−	−
8	Cream powder	B. anthracis Sterne	ND	+	+	−
9	Soil	B. anthracis	2 × 10^4^ CFU/g	+	+	+
10	Soil	B. anthracis	2 × 10^5^ CFU/g	+	+	+
11	Soil	B. anthracis	2 × 10^7^ CFU/g	+	+	+
12	Soil	B. thuringiensis	10 mg/g	−	−	−
13	Soil	Vaccinia virus	2 × 10^7^ CFU/g	−	−	−
14	Soil	F. tularensis	2 × 10^7^ CFU/g	−	−	−
15	Soil	B. pseudomallei	2 × 10^7^ CFU/g	−	−	−
16	Soil	F. tularensis plus B. pseudomallei	2 × 10^6^ CFU/g each	−	−	−

a Nucleic acid extracts from these samples were investigated in the frame of SIBCRA exercises. Laboratory results were positive for all agents other than B. anthracis (i.e., vaccinia virus, F. tularensis, B. pseudomallei, and mixed F. tularensis and B. pseudomallei).

b ND, not determined.

c To confirm that DNA in the extract was amplifiable, samples were assessed using a 16S rRNA PCR.

d −, not detected; +, detected.

### Field testing.

The BA_5345 and *adk* RPA assays were finally assessed with 13 swab samples collected and processed under field conditions in a laboratory tent. Data were compared to those obtained with a duplex qPCR assay for detection of B. anthracis *purA* SNPs and the B. cereus group *ptsI* common marker (28) (Table 4). All swab samples, except for the positive control, were negative with the BA_5345 RPA and *purA* qPCR assays (Table 4). Results obtained with the *adk* RPA assay were identical to those generated by the *ptsI* qPCR assay except for sample 2, which was RPA assay positive but qPCR assay negative (Table 4); the latter turned positive when diluted extracted DNA was tested.

**TABLE 4:** Swab samples tested in the field (PIONEX exercise, Pionki, Poland)

Sample no.	Type	Scenario	RPA results^*a*^		qPCR results^*a*^	
			BA_5345	*adk*	*purA*	*ptsI*
Control 1	NTC		−	−	−	−
Control 2	Extraction		−	−	−	−
Control 3	B. anthracis 9602		+	+	+	+
1	Swab	1	−	+	−	+
2	Swab	1	−	+	−	−
3^*b*^	Swab	1	−	−	−	−
4^*b*^	Swab	2	−	−	−	−
5	Swab	2	−	+	−	+
6	Swab	2	−	+	−	+
7	Swab	2	−	+	−	+
8	Swab	2	−	+	−	+
9	Swab	2	−	+	−	+
10	Swab	2	−	+	−	+
11	Swab	2	−	+	−	+
12	Swab	2	−	+	−	+
13	Swab	2	−	+	−	+

a −, not detected; +, detected.

b Extracted swab samples were positive when assessed using the 16S rRNA PCR.

### Blind testing at the Institute of Veterinary Bacteriology.

The three B. anthracis-specific RPA assays were carried out with a panel of DNA and lysate samples (*n* = 30, including 20 B. anthracis samples and 10 B. cereus group samples) provided by the Institute of Veterinary Bacteriology (Bern, Switzerland) and were assessed in that facility. RPA assay results were 100% concordant with previous strain characterizations (Table 5). B. anthracis-specific RPA assay results remained negative for all B. cereus DNA samples, which were efficiently amplified at equivalent concentrations by PCR assays of the *panC* gene carried out in parallel with RPA assays.

**TABLE 5:** Blind testing at the Institute of Veterinary Bacteriology (Bern, Switzerland)

Strain	Sample type	Species^*a*^	RPA result^*b*^			Identification
			BA_5345	*lef* (pXO1)	*capA* (pXO2)	
JF3788	DNA extract	B. anthracis	+	+	+	B. anthracis
JF3786	DNA extract	B. anthracis	+	+	+	B. anthracis
JF3852	DNA extract	B. anthracis	+	+	+	B. anthracis
JF3787	DNA extract	B. anthracis	+	+	+	B. anthracis
JF3785	DNA extract	B. anthracis	+	+	+	B. anthracis
JF3784	DNA extract	B. anthracis	+	+	+	B. anthracis
JF3853	DNA extract	B. anthracis	+	+	+	B. anthracis
JF3854	DNA extract	B. anthracis	+	+	+	B. anthracis
JF3783	DNA extract	B. anthracis	+	+	+	B. anthracis
JF3960	Lysate	B. anthracis	+	+	+	B. anthracis
JF3965	Lysate	B. anthracis	+	+	+	B. anthracis
JF3966	Lysate	B. anthracis	+	+	+	B. anthracis
JF3963	Lysate	B. anthracis	+	+	+	B. anthracis
JF3961	Lysate	B. anthracis	+	+	+	B. anthracis
Chad A1	Lysate	B. anthracis	+	+	+	B. anthracis
Chad A3	Lysate	B. anthracis	+	+	+	B. anthracis
Chad A5	Lysate	B. anthracis	+	+	+	B. anthracis
Chad A7	Lysate	B. anthracis	+	+	+	B. anthracis
Chad A10	Lysate	B. anthracis	+	+	+	B. anthracis
Chad A12	Lysate	B. anthracis	+	+	+	B. anthracis
JF4875^*c*^	DNA extract	B. cereus	−	−	−	B. cereus
JF5512^*c*^	DNA extract	B. cereus	−	−	−	B. cereus
JF4075^*c*^	DNA extract	B. cereus	−	−	−	B. cereus
M2841^*c*^	DNA extract	B. cereus	−	−	−	B. cereus
JF5881^*c*^	DNA extract	B. cereus	−	−	−	B. cereus
JF3778^*c*^	DNA extract	B. cereus	−	−	−	B. cereus
JF1887^*c*^	DNA extract	B. cereus	−	−	−	B. cereus
M2089^*c*^	DNA extract	B. cereus	−	−	−	B. cereus
JF4090^*c*^	DNA extract	B. cereus	−	−	−	B. cereus
JF4059^*c*^	DNA extract	B. cereus	−	−	−	B. cereus

a Genomic DNA was characterized by real-time PCR TaqMan assays targeting *sap* (chromosomal), *cap* (pXO2), and *pag* (pXO1) genes.

b −, not detected; +, detected.

c Endpoint PCR of the *panC* gene confirmed the presence of amplifiable DNA in all negative samples (B. cereus group).

## DISCUSSION

In the event of a natural, accidental, or deliberate dispersal of B. anthracis spores, there is a crucial need for fast and reliable screening and detection of contaminated patients, animals, environments, or surfaces, to immediately initiate appropriate countermeasures (29, 30). Overwhelming data published so far in this specific research area point out the lack of methods readily usable by first responders for on-site detection of B. anthracis spores, as well as the need for assays based on amplification of sequences from the pXO1 and pXO2 virulence plasmids combined with specific chromosomal markers (9, 10). In the current study, isothermal amplification and detection were carried out on a set of DNA targets including pXO1 (*lef*) and pXO2 (*capA*) plasmid virulence targets combined with BA_5345, which is one of the most stable and specific chromosomal markers identified (31, 32). It is noteworthy that a panel of 10 RPA assays was previously tested for detection of biothreat agents, including B. anthracis (19). However, the focus was on assay performance, while B. anthracis detection was restricted to the two virulence plasmids. The aim of the current study was to design a set of field-portable RPA assays usable in rapid diagnostic tests (RDTs), point-of-care testing (POCT), or point-of-need (PAN) testing. This multitarget approach allowed fast and specific screening of samples, with the detection of B. anthracis and its discrimination from closely related species belonging to the Bacillus cereus sensu lato group.

The current RPA assays were as sensitive as conventional qPCR assays, with the LOD for each target gene being as low as 10 genome-equivalent copies. In terms of sensitivity and specificity, the current assays correctly identified all B. anthracis samples (11/11 samples) among a panel of 60 reference DNAs, including species closely related to B. anthracis, with no cross-detection. Regarding the latter, it is of note that this panel included two very close phylogenetic neighbors of B. anthracis that we recently isolated from a soil sample in Namibia and characterized by whole-genome sequencing (L. M. Irenge, J. Ambroise, A.-S. Piette, B. Bearzatto, and J.-L. Gala unpublished data). Moreover, the current assays correctly identified soil and powder samples containing B. anthracis spores. Finally, swab samples contaminated with B. thuringiensis (used as a B. anthracis simulant) were rapidly and correctly identified under field conditions using a previous version of these RPA assays based only on BA_5345 and *adk* DNA targets.

Interestingly, as requested for a first screening and detection test, the RPA reactions were completed in less than 4 to 7 min, depending on the target. This is significantly faster than conventional qPCR methods with fluorescent probes, which have hands-off turnaround times of ∼1.5 h. It is also faster than other isothermal amplification methods, e.g., LAMP, which requires <45 min for signal build-up (15, 19, 33). Moreover, RPA assays can be performed on a small (74 by 178 by 188 mm), lightweight (1 kg), and user-friendly portable device. Moreover, RPA kits are now commercially available in lyophilized format. Altogether, these assay characteristics make RPA assays particularly suitable for in-field screening of suspicious materials such as white powders and soil samples in suspected areas and for the detection of B. anthracis-contaminated samples or surfaces. For these purposes, current assays may well be carried out in a van equipped with a glove box for sample inactivation and DNA extraction, as well as a dedicated space for performing analytical work and data processing. We think that such a lean organization based on an ultra-light deployable laboratory with simple, fast, and robust screening tests would enable first responders to rapidly complete a first assessment, thus saving precious time and resources in cases of white-powder hoaxes. Recent reports highlighted the feasibility of this RPA-based RDT or POCT concept for detecting Leishmania donovani using a suitcase laboratory (13), Ebola virus in Guinea (14), dengue virus (15), chikungunya virus (11), or enteric viruses (12). Further work is now required to validate the current set of RPA assays with a broader panel of soil and powder samples contaminated both with B. anthracis spores and with closely related species and to validate these assays during on-site operations in the framework of international exercises.

Initial *adk* RPA tests carried out with earlier TwistAmp exo kit batches appeared to be very specific, including when performed on-site during the exercise in Pionki, as reported in this work. Results showed unambiguously that *pst1* qPCR and *adk* RPA assays carried out under field conditions provided similar results except for sample 2, for which positivity was detected only with the RPA assay. The latter result underscored the occurrence of PCR inhibition leading to a false-negative result from the *pst1* qPCR assay for that particular sample. The RPA assay result obtained for the latter sample indicated that this isothermal amplification assay was more robust and less prone to matrix-induced inhibition effects than was the qPCR assay (34). The potential of the *adk* target gene to discriminate B. cereus group strains from bacteria of other genera, including Bacillus sp. strains, was also confirmed with a SYBR green qPCR assay using the same primers as for the *adk* RPA assay (Table 2). Despite these very promising initial RPA assay results, however, artifactual amplification in NTC samples hampered further experiments (see the supplemental material).

In conclusion, three B. anthracis-specific RPA assays were developed for rapid and reliable screening of samples commonly suspected to be contaminated by B. anthracis spores, i.e., suspicious white powders and soils, by first responders under field conditions.

## MATERIALS AND METHODS

### Bacterial strains and environmental samples.

A first panel of 60 reference bacterial strains, including purified genomic DNA from 11 B. anthracis strains, 30 B. cereus sensu lato group strains, 9 Bacillus sp. strains, and 10 Gram-positive or Gram-negative strains of other genera, as well as human DNA, was used in this study. These purified genomic DNAs were obtained from the Centre d'Etudes du Bouchet (Vert-le-petit, France), the Bundeswehr (Germany), and the collection of the Microbiology Unit of the Université catholique de Louvain (Brussels, Belgium). The latter institution also kindly provided 22 additional strains of the Bacillus cereus sensu lato group and 8 Bacillus sp. strains. Genomic DNAs of this panel of strains were characterized for the presence or absence of the virulence plasmids pXO1 and pXO2 using a previously described duplex qPCR assay (28).

A second sample collection tested in this study consisted of 8 soil samples and 8 reconstituted powder samples. Soil samples (2 g) were spiked with B. anthracis Ames strain, vaccinia virus, B. thuringiensis, Francisella tularensis Schu4 strain, Burkholderia pseudomallei, or a mixture of Francisella and Burkholderia, at concentrations between 2 × 10^4^ and 2 × 10^7^ CFU/g of soil. Powder samples, including DiPel biological insecticide, yeast, cream, and baking soda, were either spiked or not with B. anthracis Sterne strain. Prior to spiking, which was carried out at the U.S. Army Dugway Proving Ground facility, all biological agents were inactivated with cobalt gamma irradiation. These environmental samples were obtained in the frame of round-robin sample identification of biological agents (SIBA) exercises organized in February 2006 (soil samples) and January 2009 (powder samples) by the Biotesting and Production Group, West Desert Test Center, Life Science Test Facility, Dugway Proving Ground, for testing within the NATO Army Armaments Group Joint Capability Group on Chemical, Biological, Radiological, and Nuclear (CBRN) Defense, Subgroup on Sampling and Identification of Biological, Chemical, and Radiological Agents (SIBCRA).

Another set of samples consisted of 13 swabs collected and analyzed on site during the PIONEX exercise organized in the framework of the European Commission 7th Framework Program, Preparedness and Resilience against CBRN Terrorism using Integrated Concepts and Equipment, on 22 to 25 April 2014 in Pionki, Poland (35). One of the objectives of the exercise was to test and to validate RDTs usable under field conditions. In that regard, an inflatable tent laboratory was deployed close to the scene, to carry out DNA-based microbial identification using qPCR and RPA assays (Fig. 1A to F). Two scenarios were executed by the Polish State Firemen CBRN sampling team, as follows: scenario 1, swabbing barrels with suspicious substances found in a nearby forest; scenario 2, swabbing surfaces contaminated by white powders after intentional release inside a city building. After on-site collection, samples were quickly delivered to the laboratory. In scenario 1, swab samples (Table 4, samples 1 to 3) were processed for testing of the presence of five different pathogens, including the B. anthracis simulant. In scenario 2, swab samples (Table 4, samples 4 to 13) were processed for testing of the presence of B. anthracis. In both scenarios, spores of B. thuringiensis subsp. aizawai, strain ABTS-1857 (XenTari biological insecticide; Valent BioSciences, Walnut Creek, CA, USA), were used as a B. anthracis simulant. Simulant and guidelines for contamination of both scenes were provided by the Centre de Technologies Moléculaires Appliquées (CTMA)/Université catholique de Louvain laboratory staff, and samples were anonymized by the PIONEX exercise controller.

The last validation of the RPA assays was carried out in the Department of Infectious Diseases and Pathobiology, Institute of Veterinary Bacteriology (Bern, Switzerland), where all RPA assay reagents were shipped before two scientists from the CTMA (M.B. and J.-L.G.) were hosted to carry out the RPA assays. DNA samples from the Institute of Veterinary Bacteriology collection were prepared for blind testing by a local staff member (P.P.). DNA samples included B. anthracis (*n* = 20) and B. cereus group members (*n* = 10) (36–38). B. anthracis strains were previously characterized for the B. anthracis markers *sap*, *cap* (pXO2), and *pag* (pXO1) (39). B. cereus DNAs were assessed in parallel by the local scientist (P.P.), using an endpoint PCR assay targeting the *panC* gene (40).

### DNA extraction.

All strains were cultivated in the CTMA laboratory except for B. anthracis; cultivated strains were extracted using the fully automated EZ1 system (Qiagen, Venlo, The Netherlands). DNA was extracted from powder samples and swabs using the NucliSens miniMag semiautomated apparatus (bioMérieux, Boxtel, The Netherlands). DNA from spiked environmental soil samples was extracted using the PowerMax Soil DNA isolation kit (Mo Bio Laboratories, Carlsbad, CA, USA). Extraction was carried out according to the manufacturer's instructions; the resulting DNA was eluted in Tris-HCl buffer (pH 8) and stored at −20°C. The DNA extractions performed at the Institute of Veterinary Bacteriology in Bern were performed with the DNeasy Blood & Tissue kit (Qiagen, Hilden, Germany), according to the manufacturer's instructions. Lysates were carried out as described previously (38).

### RPA assay design.

Three RPA assays were designed to detect and to identify B. anthracis with high specificity, to distinguish it from closely related B. cereus pathogenic and nonpathogenic strains. These assays targeted the specific B. anthracis genomic marker BA_5345 (GenBank accession no. CP009981), together with the *lef* (GenBank accession no. CP009980) and *capA* (GenBank accession no. CP009979) genes, located on the pXO1 and pXO2 virulence plasmids, respectively. A fourth assay targeted a highly conserved region of the *adk* gene (GenBank accession no. CP009981), which is common to B. cereus group strains, as deduced from a multiple-sequence alignment performed with *adk* sequences available in GenBank (41, 42). The sequences and localization of primers and probes used for each RPA and the expected sizes of the RPA amplicons are depicted in Table 1. Positions were numbered according to the reference genomic sequence of B. anthracis strain Ames BA1004 (Table 1). The lengths of RPA primers and probes were set to 30 to 35 nucleotides and ∼45 nucleotides, respectively. Each probe was designed by introducing a tetrahydrofuran (THF) abasic nucleotide analogue at position 30, flanked by the dT-5-carboxyfluorescein (FAM) fluorophore and the dT-black hole quencher 1 (BHQ1) quencher at positions 28 and 32, respectively (Table 1). RPA primers and probes were designed based on rules and guidelines provided by the manufacturer and were purchased from Eurogentec (Liège, Belgium). The specificity of the primers and probes for their specific targets was studied *in silico* using the BLAST search tool and the Biostrings R package. The latter was used to identify perfect matches with the whole-genome sequences of B. anthracis and closely related species (B. cereus, B. thuringiensis, B. mycoides, B. cytotoxicus, and B. weihenstephanensis), which are available in GenBank (total *n* = 171).

### Optimization of RPA assay conditions.

Initial optimization was carried out using the TwistAmp Basic kit (TwistDx Ltd., Cambridge, UK). A gel electrophoresis analysis was carried out after amplification with tested primers, in order to improve the amplification yield through selection of optimal target sequences. The final set of primers was selected based on the greatest amplification efficiency and negative or lowest NTC value. After the selection of optimal target regions, a second phase of assay optimization was carried out using the real-time fluorescent DNA amplification TwistAmp exo kit from the same manufacturer. The RPA reaction was performed in a final volume of 50 μl containing 420 nM each RPA primer, 120 nM RPA probe (except for the *lef* probe, which was used at 60 nM), 14 mM magnesium acetate, and 1× rehydration buffer. All reagents except the tested DNA sample and magnesium acetate were prepared in a master mix, which was distributed into 0.2-ml reaction tubes containing a dried enzyme pellet. A volume of 5 μl of sample DNA (5 pg) or control standard was added to the tube. Next, magnesium acetate was pipetted into the tube lids. The lids were closed, the magnesium acetate was centrifuged into the tubes using a minispin centrifuge, and the tubes were immediately placed in a Twista portable real-time fluorometer (TwistDx). Fluorescence measurements in the FAM channel were carried out every 20 s for 20 min, at 42°C. To distinguish positive from negative results, a cutoff value was calculated for every individual sample according to the guidelines on threshold validation in the Twista Studio software manual. A sample was deemed positive if all replicates were >3 standard deviations above the background during a defined time period (i.e., after 19 to 20 min of amplification). Slope validation was also carried out according to the guidelines.

### Real-time PCR.

Two duplex qPCR assays described previously were used to identify B. anthracis and to distinguish B. cereus group strains from other species (28). The first qPCR assay was based on *purA* and *ptsI* targets, while the second allowed detection of the B. anthracis virulence plasmids pXO1 and pXO2. A SYBR green qPCR assay was developed to detect the *adk* target gene. This qPCR assay was carried out in a 25-μl reaction volume containing 12.5 μl of 2× power SYBR green master mix (Life Technologies, Carlsbad, CA, USA), 165 nM each primer, and 2.5 μl of tested genomic DNA. The reaction was initiated at 50°C for 2 min and 95°C for 10 min, followed by 45 cycles of denaturation at 95°C for 15 s, annealing at 63°C for 1 min, and extension at 72°C for 90 s. Each sample was tested in triplicate, and data were recorded as cycle threshold (*C_T_*) values on a Bio-Rad CFX96 detection system (Bio-Rad Laboratories N.V., Temse, Belgium), using the analytical software from the same manufacturer.

### Plasmid DNA and calibration curves.

The *lef*, *capA*, BA_5345, and *adk* genes were amplified by PCR using specific pairs of primers (Table 1) and genomic DNA from the B. anthracis 9774 strain. Primers were designed using the Primer3 algorithm (43). Each PCR mixture (50 μl) contained 1 ng of genomic DNA, 250 nM each primer, 250 μM each deoxynucleoside triphosphate (dNTP), 1.5 mM MgCl_2_, 0.7 U/reaction AmpliTaq polymerase, and 1× AmpliTaq buffer II (Roche Molecular Systems, Branchburg, NJ, USA). The thermal amplification profile consisted of a denaturation step at 94°C for 3 min followed by 35 cycles of thermal cycling at 94°C for 30 s, 55°C for 30 s, and 72°C for 45 s; after cycling, the reaction tubes were maintained at 72°C for 7 min. The PCR products were purified using the QIAquick gel extraction kit (Qiagen, Hilden, Germany), ligated into pCR4-TOPO, and transformed into One Shot TOP10 electrocompetent Escherichia coli cells using the TOPO TA cloning kit for sequencing (Life Technologies). The resulting recombinant plasmids were purified using the Invisorb Spin Plasmid Mini Two kit (Invitek, Berlin, Germany) and verified by sequencing with a BigDye Terminator v1.1 cycle sequencing kit (Applied Biosystems, USA), using an automated sequencer (3130 Genetic Analyser; Applied Biosystems). The BA_5345, *lef*, and *capA* recombinant plasmids were quantified by UV measurements with a NanoDrop ND-1000 v3.5.2 spectrophotometer (Isogen Life Science, Utrecht, The Netherlands). Calibration curves were created from 10-fold diluted recombinant plasmids with a range of 10^6^ to 10^0^ copies per reaction, as calculated from the plasmid concentrations (optical density at 260 nm). The BA_5345, *lef*, and *capA* gene plasmid standards were tested in five replicates, and the threshold time (in minutes) was plotted against the number of molecules detected.

### Statistical analysis of sensitivity.

The LOD was determined using a probit analysis. For each assay, a probit regression model was fitted with the glm function of R statistical software (http://www.r-project.org), using the target concentration as the independent variable and the detection event as the response variable. Each probit regression model was then used to determine the lowest concentration enabling target detection with a 95% probability (i.e., the LOD).

## Supplementary Material

Supplemental material
