# Supplementary material for: Sensitive and Specific Recombinase Polymerase Amplification Assays for Fast Screening, Detection, and Identification of Bacillus anthracis in a Field Setting
Source: Appl Environ Microbiol. 2018 May 17;84(11):e00506-18. doi: 10.1128/AEM.00506-18 (PMC5960963; doi:10.1128/AEM.00506-18)
Supplement: Supplemental material [file supp_84_11_e00506-18__index.html]

Supplemental material 

# Sensitive and Specific Recombinase Polymerase Amplification Assays for Fast Screening, Detection, and Identification of Bacillus anthracis in a Field Setting

## Supplemental material

- Supplemental file 1 -

  Discussion of *adk* RPA test.

  PDF, 12K
