# Supplementary material for: Sensitive and Specific Recombinase Polymerase Amplification Assays for Fast Screening, Detection, and Identification of Bacillus anthracis in a Field Setting
Source: Appl Environ Microbiol. 2018 May 17;84(11):e00506-18. doi: 10.1128/AEM.00506-18 (PMC5960963; doi:10.1128/AEM.00506-18)
Supplement: Supplemental material [file AEM.00506-18_zam011188535s1.pdf]

Discussion of *adk*-RPA test

To test the specificity of this marker on the panel of 61 reference DNAs and 16 environmental powder and soil samples, we had indeed to order new batches of the test kit which all generated previously unseen non-specific, artefactual amplification in NTC samples. Despite all our efforts, this sudden occurrence of non-specific signals could never been circumvented. A contamination of the premix laboratory environment was ruled out when no improvement was observed despite the use of new fresh reagents, repeated thorough decontaminations of laboratory rooms and equipment, and complementary experiments in another distant laboratory facility not involved the current work. Consequently, a contamination of the RPA commercial mix with DNA from B. cereus during manufacturing was postulated, despite remaining unconfirmed by the manufacturer.
